# FTY720-induced endocytosis of yeast and human amino acid transporters is preceded by reduction of their inherent activity and TORC1 inhibition

**DOI:** 10.1038/s41598-017-14124-2

**Published:** 2017-10-23

**Authors:** Céline Barthelemy, Abdoulaye Oury Barry, Laure Twyffels, Bruno André

**Affiliations:** 1 Molecular Physiology of the Cell, Université libre de Bruxelles (ULB), IBMM (Biopark), Gosselies, Belgium; 2 Center for Microscopy and Molecular Imaging (CMMI), Université libre de Bruxelles (ULB), IBMM (Biopark), Gosselies, Belgium

## Abstract

FTY720 is a sphingoid base analog that acts as an anticancer agent in animal models. Its effect on tumor cells stems largely from its ability to trigger endocytosis of several nutrient transporters. The observation that FTY720 similarly stimulates downregulation of amino acid permeases in yeast suggests that the cellular mechanisms it targets, which are still poorly characterized, are evolutionarily conserved. We here report that adding FTY720 to yeast cells results in rapid inhibition of the intrinsic activity of multiple permeases. This effect is associated with inhibition of the TORC1 kinase complex, which in turn promotes ubiquitin-dependent permease endocytosis. Further analysis of the Gap1 permease showed that FTY720 elicits its ubiquitylation via the same factors that promote this modification when TORC1 is inhibited by rapamycin. We also show that FTY720 promotes endocytosis of the LAT1/SLC7A5 amino acid transporter in HeLa cells, this being preceded by loss of its transport activity and by mTORC1 inhibition. Our data suggest that in yeast, TORC1 deactivation resulting from FTY720-mediated inhibition of membrane transport elicits permease endocytosis. The same process seems to occur in human cells even though our data and previous reports suggest that FTY720 promotes transporter endocytosis via an additional mechanism insensitive to rapamycin.

## Introduction

2-Amino-2-[2-(4-octylphenyl)]-1,3-propanediol hydrochloride, also known as FTY720 or fingolimod, is a synthetic derivative of myriocin, a natural antibiotic isolated from the pathogenic fungus *Isaria sinclairii* ^1^. FTY720 is used as an immunosuppressant to treat multiple sclerosis, a common inflammatory disorder of the central nervous system^2^. The drug, structurally related to sphingoid bases, is phosphorylated *in vivo* by sphingosine kinase 2. Once phosphorylated, it can bind to G-protein-coupled sphingosine-1-phosphate (S1P) receptors^3,4^, this inducing their internalization^5^. This modulation of S1P receptors by FTY720 is associated with altered lymphocyte trafficking and immunosuppression^2,6,7^. At higher doses than required for immunosuppression, FTY720 also causes death of several types of tumor cells^8^. This effect is independent of S1P receptors and is largely due, rather, to the ability of FTY720 to promote endocytosis of several nutrient transporters, thus reducing the ability of cancer cells to meet their high anabolic demands^9^. The drug notably promotes downregulation of Cat-1 (cationic amino acid transporter 1), Glut1 (glucose transporter 1), and 4F2hc. This last, also named CD98 or SLC3A2^9^, is a transmembrane protein which associates with various transporters via a disulfide bridge and is required for their proper cell-surface secretion. One 4F2hc-associated transporter is LAT1 (« L-Type amino acid transporter 1 »), also known as SLC7A5, the large neutral amino acid transporter^10,11^. LAT1 is the main leucine transporter in most tumor cells and thus plays a key role in activation of the mTORC1 kinase complex by leucine^12–15^. Recent work has revealed that FTY720 contributes to tumor cell death via yet another mechanism: inhibition of PI(3)P 5-kinase, the enzyme producing PI(3,5)P2, through mislocalization^16^. This inhibition causes accumulation of enlarged endosomes (vacuoles) containing intraluminal vesicles, along with inhibition of autophagosome formation and autophagosome-lysosome fusion. The resulting reduction of the autophagic flux enhances the metabolic stress induced by transporter downregulation, thereby efficiently promoting tumor cell death^16^.

The mechanism underlying FTY720-induced transporter endocytosis remains poorly understood. The drug seems to act via stimulation of protein phosphatase 2A (PP2A), as PP2A inhibitors have been found to reduce FTY720-induced transporter downregulation^8,16,17^. The action mechanism of FTY720 might be evolutionarily conserved, since the drug also promotes transporter downregulation in yeast. Specifically, FTY720 is reported to cause degradation of the Tat1 tryptophan transporter, and it likely acts similarly on other permeases as well. For example, leucine uptake is reduced in FTY720-treated cells^18^. Endocytosis of yeast plasma membrane permeases is typically triggered by their ubiquitylation^19^. This modification is catalyzed by Rsp5, a ubiquitin (Ub) ligase of the Nedd4 family^20,21^, acting in association with adaptors of the α-arrestin family^19,22,23^. Amino acid substitutions altering the Ub-acceptor lysines or the presumed α-arrestin binding site of permeases confer protection against ubiquitylation and endocytosis^24–26^. The signals and pathways triggering permease ubiquitylation and downregulation are diverse: a change in the nutritional status of the cell^24,27^, a shift to stress conditions^28,29^, or the conformational changes of the permease itself coupled to transport catalysis^25,30,31^. In support of the view that FTY720-induced endocytosis of Tat1 is Ub-dependent, FTY720 has been shown to inhibit growth of tryptophan auxotrophs, this inhibition being less pronounced in yeast strains with mutations in the *RSP*5 gene or in the *BUL1* gene encoding an α-arrestin^18^.

In this study, we have further investigated the mechanisms underlying FTY720-induced endocytosis of transporters. We first show that multiple yeast permeases undergo FTY720-induced Ub-dependent downregulation. We then provide evidence that the intrinsic activity of multiple nutrient permeases is reduced upon FTY720 addition, this being associated with rapid inhibition of TORC1, which in turn promotes Ub-dependent permease endocytosis. We next show that FTY720 also triggers LAT1 endocytosis in HeLa human cells, and that this effect is preceded by a reduction of LAT1 activity and inhibition of mTORC1. We discuss models according to which transporter inactivation coupled to TORC1 inhibition could contribute importantly to transporter endocytosis in FTY720-treated yeast and human cells.

## Results

### FTY720 promotes Rsp5-dependent endocytosis of multiple permeases in yeast

**Figure 1:**
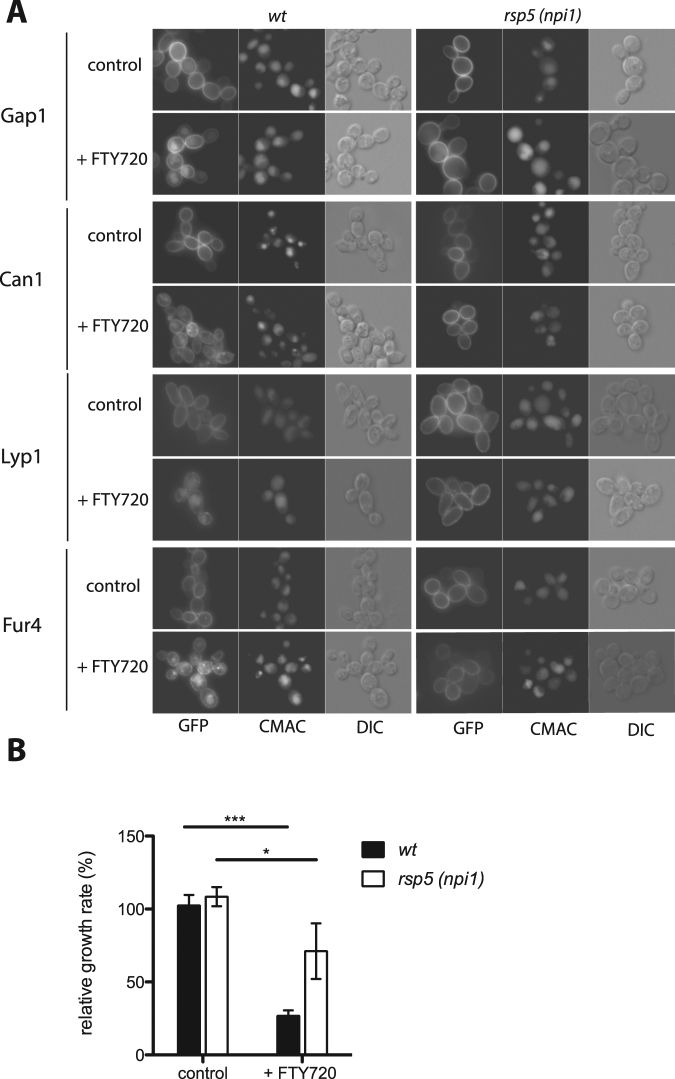
FTY720 promotes Rsp5-dependent endocytosis of multiple permeases. (**A**) Strains 23344C (*ura3*) and 27038a (*rsp5/npi1 ura3*) transformed with plasmid pJOD10 (YCpGAL-GAP1-GFP), pCJ563 (YCpGAL1-CAN1-GFP), pNAM001 (YCp-LYP1-GFP), or pFL38-gF-GFP (YCpGAL1-FUR4-GFP) were grown on galactose-proline (Gap1-GFP, Can1-GFP, and Lyp1-GFP cells) or galactose ammonium (Fur4-GFP cells) medium. Glucose was added for 90 min before addition of FTY720 or DMSO solvent alone (control). Cells were examined by wide field microscopy after a 2-h incubation. Cells were also labeled with CMAC dye to visualize the vacuole. (**B**) Strains 23344C (*ura3*) and 27038a (*rsp5/npi1 ura3*) transformed with an empty *URA3* plasmid (pFL38) were grown on glucose ammonium liquid medium. FTY720 was added to half of the cultures. Relative growth rates were measured using strain 23344c in the FTY720-free medium as a reference (control). Values represent the means of three independent experiments and error bars correspond to standard deviations (SD) (unpaired *t*-test, *P < 0.05, ***P < 0.001).

FTY720 inhibits the growth of various types of cancer cells by simultaneously stimulating endocytosis of plasma-membrane nutrient transporters and inhibiting lysosomal fusion and function, thereby causing nutrient limitation and finally cell death^16^. Although PP2A phosphatases appear to play an important role in the cellular effects of FTY720, the molecular mechanisms involved in FTY720-induced transporter downregulation remain elusive. According to a previous study, FTY720 also promotes Ub-dependent degradation of the yeast Tat1 permease^18^. This prompted us to investigate the mechanisms of FTY720-induced transporter downregulation in the yeast model system. We first examined the influence of FTY720 on the subcellular location of four GFP-fused yeast permeases: the general amino acid permease Gap1, the arginine permease Can1, the lysine permease Lyp1, and the uracil permease Fur4. Before FTY720 addition, all four transporters accumulated stably at the cell surface (Fig. 1A). Interestingly, upon FTY720 addition, each protein was found to undergo downregulation, as judged by its internalization and partial colocalization with CMAC dye used to stain the lumen of the vacuole (Fig. 1A). In contrast, all permeases remained stable at the plasma membrane upon FTY720 treatment in the *rsp5*(*npi1*) mutant, where expression of the Rsp5 ubiquitin ligase is greatly reduced^20^. These results show that FTY720 promotes Ub-dependent endocytosis of multiple plasma membrane transporters. This process should reduce nutrient uptake and impair growth. Accordingly, FTY720 was found to inhibit growth of a prototrophic yeast strain on minimal medium. In keeping with previous observations, the *rsp5*(*npi1*) mutant largely resisted this growth inhibition (Fig. 1B)^18^, although FTY720 still significantly reduced its growth (Fig. 1B). This suggests that FTY720 might exert other detrimental effects in addition to permease endocytosis.

### FTY720 promotes Gap1 ubiquitylation and endocytosis via the stress-responsive Bul-Aly α-arrestin system

**Figure 2:**
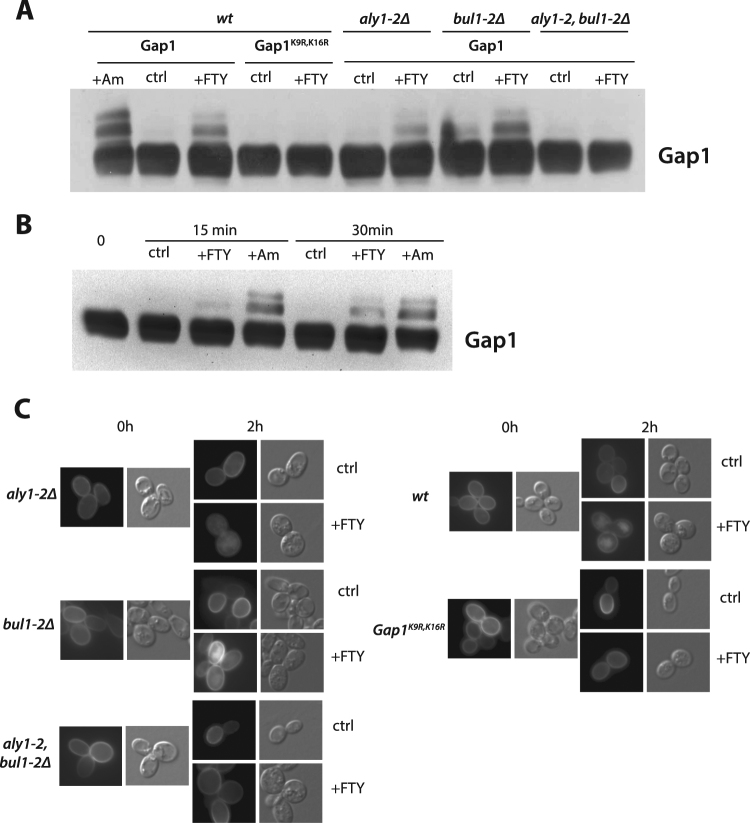
FTY720 promotes Gap1 ubiquitylation via the stress-responsive Bul-Aly α-arrestin system. (**A**) Strains EK008 (*gap1*Δ *ura3*), JA493 (*gap1*Δ *bul1*Δ *bul2*Δ *ura3*), 35101a (*gap1*Δ *aly1*Δ *aly2*Δ *ura3*), and MA062 (*gap1*Δ *bul1*Δ *bul2*Δ *aly1*Δ *aly2*Δ *ura3*) transformed with plasmid pJOD10 (YCpGAL-GAP1-GFP) or pCJ038 (YCpGAL-GAP1^K9R,K16R^-GFP) were grown on galactose-proline medium. Cells were collected before and 30 min after addition of FTY720 (+FTY). Ammonium (50 mM) was also added for 15 min (+Am) to *gap1*Δ *ura3*/YCpGAL-GAP1-GFP cells, as a positive control for Gap1 ubiquitylation. Crude cell extracts were immunoblotted with anti-GFP antibody. (**B**) Strain EK008 (*gap1*Δ *ura3*) transformed with plasmid pJOD10 (YCpGAL-GAP1-GFP) was grown on galactose-proline medium. Cells were collected before and 15 or 30 min after addition of DMSO (ctrl, control), FTY720 (+FTY), or ammonium (+Am). Crude cell extracts were prepared and immunoblotted with anti-GFP antibody. (**C**) Strains as in A, transformed with plasmid pJOD10 (YCpGAL-GAP1-GFP), were grown on galactose-proline medium. Glucose was added for 90 min before addition of FTY720 (+FTY) or DMSO solvent alone (ctrl, control). Cells were examined by wide field microscopy before and 2 h after FTY720 or DMSO addition.

That FTY720 triggers Rsp5-dependent endocytosis of permeases suggests that the drug can elicit their ubiquitylation. To test this, Gap1 was used as a model system. FTY720 was added to cells growing on minimal medium and cell extracts were prepared and analyzed on immunoblots. FTY720 addition caused the appearance of two upper bands above the immunodetected Gap1 signal (Fig. 2A). These bands were similar to those detected upon ammonium addition, well known to trigger Gap1 ubiquitylation^24^. Furthermore, they were not detected when the two Ub acceptor lysines of Gap1 present in the N-terminal tail (at positions 9 and 16) were replaced with arginines (Fig. 2A). We conclude that FTY720 triggers Gap1 ubiquitylation.

Previous studies have shown that different pathways can promote Gap1 ubiquitylation. One involves activation of the TORC1 kinase complex, which stimulates by dephosphorylation the redundant Bul1 and Bul2 α-arrestin-type adaptors of the Rsp5 Ub ligase^24^. The conformational changes of Gap1 during transport catalysis also elicit Gap1 ubiquitylation. This conformation-dependent Gap1 ubiquitylation also depends on the Bul α-arrestins, which in this case need not be stimulated by TORC1^25^. Lastly, Gap1 ubiquitylation and downregulation can also result from direct inhibition of TORC1 by rapamycin and from various stress conditions known or assumed to inhibit TORC1. This pathway involves the Aly1 and Aly2 α-arrestins as well as Bul1 and Bul2, as complete loss of Gap1 ubiquitylation upon TORC1 inhibition is observed only in the *bul1 bul2 aly1 aly2* quadruple mutant strain^32^. To determine which pathway promotes FYT720-induced Gap1 ubiquitylation, we first assessed the role of the Bul proteins (Fig. 2A,C). FTY720 was found to induce efficient Gap1 ubiquitylation and endocytosis in the *bul1 bul2* strain, but not in the *bul1 bul2 aly1 aly2* mutant, where Gap1 was not ubiquitylated and remained stable at the cell surface. In the *aly1 aly2* strain, Gap1 was ubiquitylated and targeted to the vacuole in response to FTY720, but less efficiently than in the wild type (Fig. 2A,C). These results show that Aly1/2 and, to a lesser extent, Bul1/2 promote FTY720-induced Gap1 ubiquitylation and downregulation. They further suggest that FTY720 elicits Gap1 ubiquitylation via the stress pathway.

### TORC1 is rapidly inhibited in FTY720-treated yeast cells

**Figure 3:**
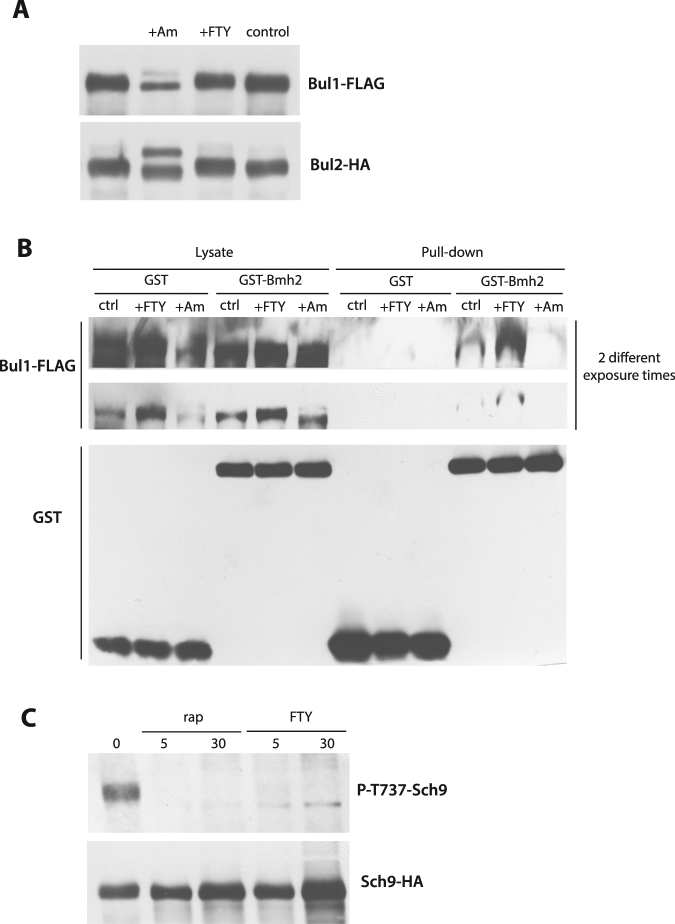
TORC1 is rapidly inhibited in FTY720-treated cells. (**A**) Strains MA025 (*gap1*Δ *bul2*Δ *BUL1-FLAG ura3*) and MA032 (*gap1*Δ *BUL2-HA ura3*) transformed with plasmid pJOD10 (YCpGAL-GAP1-GFP) were grown on galactose-proline medium. Ammonium (50 mM) (+Am), FTY720 (10 μM) (+FTY), or DMSO (control) was added for 30 min. Crude cell extracts were immunoblotted with anti-FLAG or anti-HA antibodies. (**B**) Strain MA025 (*gap1*Δ *bul2*Δ *BUL1-FLAG ura3*) transformed with plasmid pRS426-GST or pRS426-GST-BMH2 was grown on glucose proline medium. Cells were collected before and 30 min after ammonium (50 mM) (+Am) or FTY720 (10 μM) (+FTY) addition. The cells were lysed and GST was pulled down as described under Materials and Methods. Lysates and pulldown fractions were immunoblotted with anti-GST or anti-FLAG antibodies. (**C**) Strain 23344c (*ura3*) transformed with plasmid pl436 (HA-Sch9) was grown on glucose proline medium. Rapamycin (200 ng/ml) (rap) or FTY720 (10 μM) (FTY) was then added for 5 or 30 min. Crude cell extracts were immunoblotted with anti- P-T737-Sch9 and anti-HA antibodies.

In cells grown on a poor nitrogen source such as proline, the TORC1 kinase complex is only moderately active. The Npr1 kinase (inhibited by TORC1) is therefore active and phosphorylates the Bul proteins, causing their inhibitory association with the 14-3-3 phosphobinding proteins. Upon addition of ammonium (a good nitrogen source stimulating TORC1 and thereby inhibiting Npr1), the Bul proteins are activated via dephosphorylation and dissociation from the 14-3-3s, and a fraction of the dephosphorylated Buls are monoubiquitylated^24^. These post-translational changes are typically reflected on immunoblots by splitting of the initial Bul1 or Bul2 signal into two separate bands, a lower band corresponding to the dephosphorylated form and an upper band to the dephosphorylated and ubiquitylated form^24^. These changes in migration were not observed after addition of FTY720 (Fig. 3A). Dissociation of the Bul proteins from the 14-3-3s, monitored in a GST pulldown assay, was observed in ammonium-treated but not FTY720-treated cells (Fig. 3B). These results suggest that when the Bul proteins mediate FTY720-induced ubiquitylation of Gap1, they remain phosphorylated and largely bound to the 14-3-3s. As this situation is very similar to that previously observed in proline-grown cells upon TORC1 inhibition by rapamycin^29^, we suspected that TORC1 might be inhibited in the presence of FTY720. This inhibition should result in higher activity of the Npr1 kinase and thus in more pronounced Bul1 phosphorylation. Accordingly, the amount of Bul1 pulled down by the GST-fused 14-3-3 protein was significantly increased in FTY720-treated cells (Fig. 3B). To assess TORC1 inhibition in FTY720-treated cells, we monitored the phosphorylation status of the Sch9 kinase, a major TORC1 substrate. For this we used an antibody directed against a short Sch9 peptide including the phosphorylated residue Thr737, which is targeted by TORC1^33^. We grew cells on minimal proline medium, added FTY720, incubated the cells in its presence for 5 or 30 min, and then immunodetected phosphorylated Sch9 in cell extracts. As a control, we also analyzed cells treated with rapamycin. In response to rapamycin, Sch9 was dephosphorylated, as expected. A similar result was observed in FTY720-treated cells, indicating that TORC1 is inhibited in the presence of FTY720 (Fig. 3C).

### FTY720 reduces the intrinsic activity of multiple permeases stabilized at the plasma membrane

Inhibition of TORC1 in FTY720-treated cells could in principle result from nutrient starvation due to downregulation of multiple permeases. Yet in the presence of FTY720, ubiquitylation and endocytosis of Gap1 were not obvious until ∼30 min after FTY720 addition (Fig. 2B), whereas TORC1 seemed largely inhibited after only 5 min (Fig. 3C). As direct inhibition of TORC1 by rapamycin also results in relatively slow Ub-dependent endocytosis of multiple permeases^29^, we hypothesized that FTY720 might act similarly: rapid suppression of TORC1 signaling by FTY720 might in turn promote slower, Ub-dependent permease downregulation. As FTY720 is structurally similar to sphingoid bases, an immediate effect of the drug might be to impede the intrinsic activity of several plasma membrane permeases. The resulting reduction of nutrient uptake could cause rapid TORC1 inactivation, which in turn would trigger gradual Ub-dependent permease downregulation.

**Figure 4:**
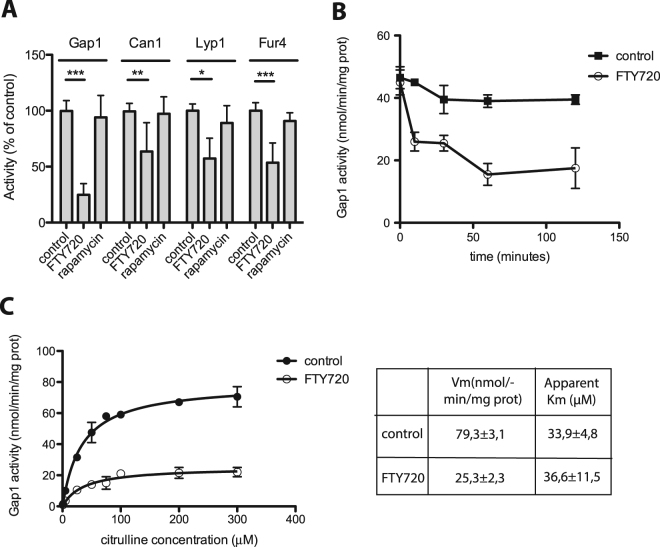
FTY720 affects the activity of various permeases. (**A**) Strains 27038a (*rsp5*(*npi1*) *ura3*), CJ005 (*gap1*Δ *rsp5*(*npi1*) *ura3*), and 35288a (*gap1*Δ *can1Δ rsp5*(*npi1*) *ura3*) were transformed with plasmid pFL38 (URA3). Cells were treated or not with FTY720 (10 μM) or rapamycin (200 ng/ml) for 1 h. The initial uptake rate for 75 μM [^14^C]-labeled citrulline (reflecting Gap1 activity), 10 μM [^14^C]-labeled arginine (reflecting Can1 activity), 10 μM [^14^C]-labeled lysine (reflecting Lyp1 activity), or 5 μM [^14^C]-labeled uracil (reflecting Fur4 activity) was then measured. Error bars correspond to SD, n = 3–5 (unpaired *t*-test, *P < 0.05, **P < 0.01, ***P < 0.001). (**B**) Strain EK008 (*gap1*Δ *ura3*) transformed with plasmid pCJ038 (YCpGAL-GAP1^K9R,K16R^-GFP) was grown on galactose-proline medium. Glucose was added for 90 min to inhibit Gap1 synthesis. Cells were treated for 0, 10, 30, 60, or 120 min with FTY720 (10 μM final concentration) or DMSO alone (control). The initial uptake rate of [^14^C]-labeled citrulline (75 μM), reflecting Gap1 activity, was then measured. Error bars correspond to SD, n = 2. (**C**) Strain EK008 (*gap1*Δ *ura3*) transformed with pCJ038 (YCpGAL-GAP1^K9R,K16R^-GFP) was grown on galactose-proline medium. Glucose was added for 90 min to repress Gap1 synthesis. Cells were treated for 60 min with FTY720 or the solvent DMSO alone (control) and the uptake of [^14^C]-citrulline added at various concentrations was then measured. Error bars correspond to SD, n = 2.

To assess this model, we used *rsp5*(*npi1*) mutant cells, in which FTY720-induced endocytosis of permeases is impaired, to monitor the activity of the Gap1, Can1, Lyp1, and Fur4 permeases before and one hour after FTY720 addition (Fig. 4A). As hypothesized, all of the tested permeases showed reduced activity (to various degrees) in the presence of FTY720. This was not due to the TORC1 inhibition observed under these conditions, since the permeases remained active after one hour of incubation with rapamycin (Fig. 4A). We conclude that the intrinsic activity of multiple permeases is reduced in FTY720-treated cells. This effect could explain why growth of the *rsp5*(*npi1*) mutant is reduced in the presence of FTY720 (Fig. 1B).

To further analyze this effect, we grew cells expressing the non-ubiquitylable Gap1^K9R,K16R^ mutant. This mutant is resistant to FTY720-induced endocytosis. We monitored the activity of the mutant permease before and at several times after FTY720 addition. We observed a 40% decrease in Gap1 activity within 10 min and a slower drop thereafter (Fig. 4B). This might in principle reflect an altered apparent affinity (*K*_m_), an altered maximum velocity (V_max_), or both. We therefore measured the kinetic parameters of Gap1^K9R,K16R^ before and one hour after FTY720 addition, and compared the results obtained. The drug was found to reduce the V_max_ but not the apparent *K*_m_ of the permease (Fig. 4C).

We next considered the possibility that the V_max_ reduction of Gap1 in FTY720-treated cells might be caused directly by the drug embedded in the plasma membrane. For instance, we have previously found sphingolipids to be crucial for proper folding of newly synthesized Gap1^34^. As FTY720 is structurally similar to sphingoid bases, it might interact directly with Gap1 and alter its conformation. We thus tested whether FTY720 might alter the sensitivity of Gap1^K9R,K16R^ to limited trypsinolysis, having observed such a change in cells where sphingolipid biogenesis is impaired^34^. We observed no significant influence of FTY720 on the trypsin digestion pattern of Gap1^K9R,K16R^ (Fig. S1A). As a control, we heated the cell extracts at 60 °C and observed, as expected, a much higher sensitivity of Gap1^K9R,K16R^ to trypsinolysis (Fig. S1B). It could be that the FTY720-induced conformational changes of Gap1^K9R,K16R^ are too subtle to be detected with this assay. Alternatively, FTY720 might reduce the activity of Gap1 and other permeases via another mechanism. It might, for example, somehow reduce the activity of the Pma1 H^+^-ATPase, which establishes the H^+^ gradient needed for Gap1 activity. Whatever the mechanism involved, our results show that reduction of the inherent activity of multiple permeases, associated with TORC1 inhibition, is an early event following FTY720 addition.

### FTY720 promotes the endocytosis of human LAT1/SLC7A5

We next investigated further the mechanism of FTY720-induced transporter endocytosis in human cells. We chose as a model system the plasma membrane bidirectional amino-acid transporter LAT1^10^, known to catalyze the uptake of various amino acids, including leucine, in a reaction coupled to glutamine efflux^12^. Like the yeast amino acid permeases, LAT1 belongs to the APC structural family of transporters^35^. Furthermore, LAT1 associates via a disulfide bridge with the 4F2hc transmembrane chaperone protein, shown to undergo endocytosis in the presence of FTY720^8^.

**Figure 5:**
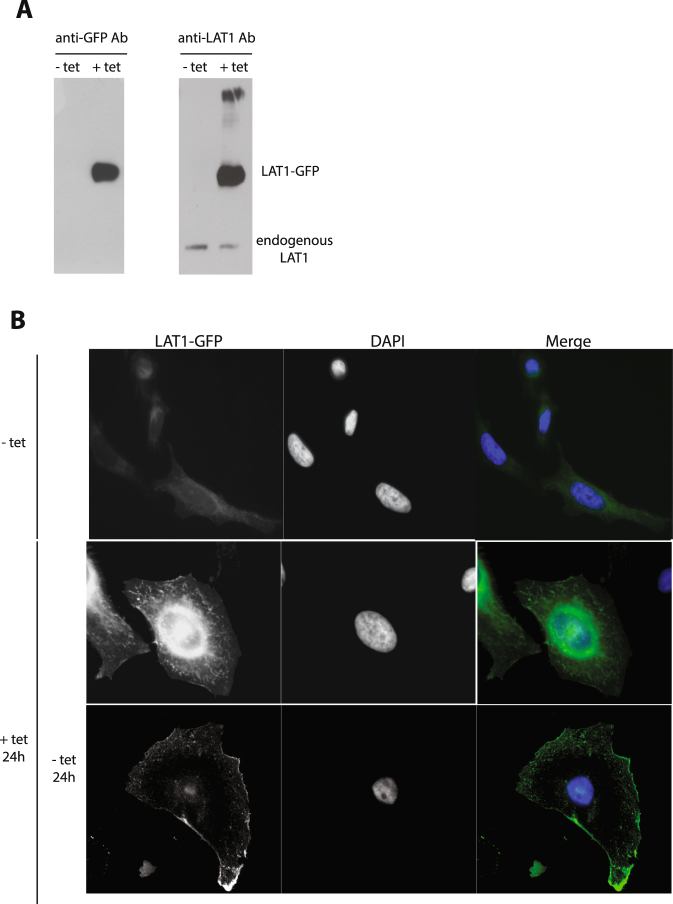
Isolation of a stable T-REx HeLa cell line expressing LAT1-GFP under the control of a tetracycline-inducible promoter. (**A**) Cells of the isolated T-REx HeLa clone were grown for 24 h in the absence (−tet) or presence (+tet) of 1 μg/ml tetracycline used to induce expression of LAT1-GFP. Cell extracts were then prepared and immunoblotted with anti-LAT1 or anti-GFP antibodies. (**B**) Cells of the same clone were grown for 24 h in the absence (−tet) or presence (+tet) of 1 μg/ml tetracycline, used to induce expression of LAT1-GFP. Part of the tetracycline-treated cells were washed and left for an additional 24 h without tetracycline. The cells were fixed and the localization of LAT1-GFP was then examined by wide field microscopy.

**Figure 6:**
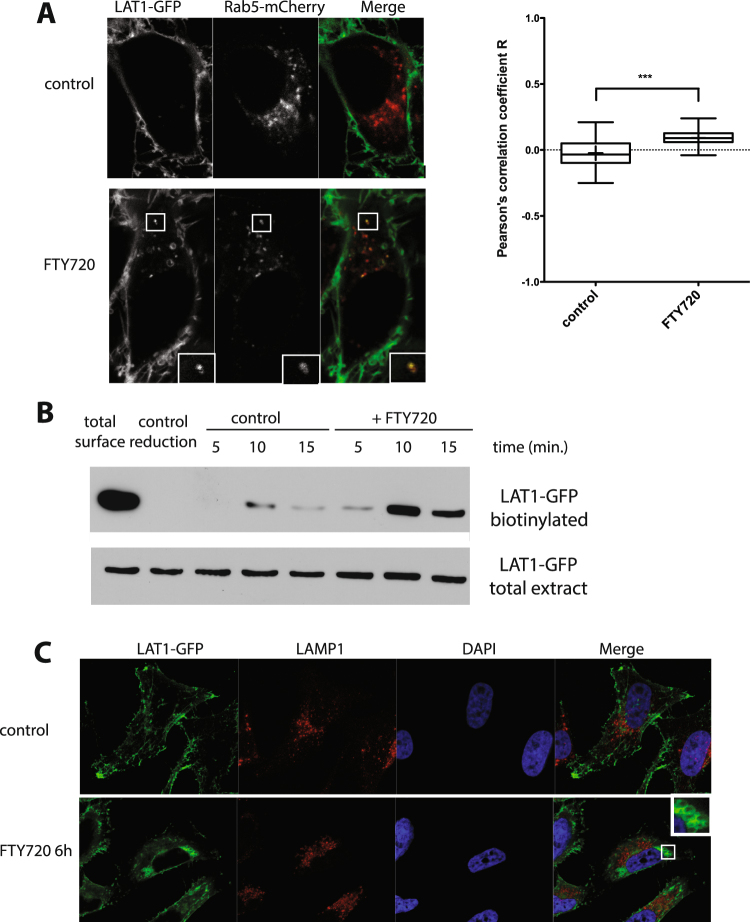
FTY720 affects LAT1 localization. (**A**) Cells of the stable T-REx HeLa line expressing LAT1-GFP were transfected with a plasmid expressing Rab5-mCherry, grown for 24 h in the presence of tetracycline, and then for an additional 40 h in tetracycline-free medium. Live cell images were acquired after 10 min of treatment with FTY720 (5 μM final concentration) or DMSO solvent alone (control). The graph corresponds to quantification of the images with Pearson’s correlation coefficient. In the boxplots, the middle line denotes the median and the top and bottom of the box indicate, respectively, the 75th and the 25th percentile. The whiskers denote the maximum and minimum values. Unpaired *t*-test ***P ≤ 0.001, n ≥ 32 cells. (**B**) Cell-surface biotinylation assay for monitoring FTY720-induced LAT1-GFP endocytosis. Cells of the stable T-REx HeLa line expressing LAT1-GFP were grown as in A and incubated with sulfo-NHS-biotin for 30 min at 4 °C. The cells were then incubated in the presence of FTY720 (5 µM final concentration) or the DMSO solvent alone (control) for the indicated times at either 4 °C to block membrane trafficking (control reduction) or 37 °C. The cells were washed and the remaining surface biotin was then cleaved except in a control sample (total surface). Biotinylated proteins were purified by affinity chromatography with streptavidin-coated beads. LAT1-GFP was then detected by immunoblotting with anti-GFP antibody. A blot representative of three independent experiments is shown. (**C**) Cells of the stable T-REx HeLa line expressing LAT1-GFP were grown as in A. They were then treated for 6 h with DMSO (control) or 5 μM FTY720. The cells were fixed and the localization of LAT1-GFP and LAMP1 was examined by confocal microscopy. Nuclei were stained with DAPI.

We generated a stable T-REx HeLa cell line expressing a LAT1-GFP fusion protein under the control of a tetracycline-regulated promoter (Fig. 5). In the presence of tetracycline, the induced LAT1-GFP protein was detected both at the cell surface and in internal membranes corresponding to the secretory pathway. After removal of the antibiotic for 24 hours, the presynthesized LAT1-GFP was detected mainly at the plasma membrane (Fig. 5B). These conditions of transient induction were thus suitable for testing whether FTY720 promotes LAT1-GFP endocytosis (Fig. 6A). Adding FTY720 did cause partial redistribution of cell-surface LAT1-GFP into small internal compartments visible in live cells. A significant proportion of these compartments could be labeled with the mCherry-Rab5 marker, indicating that they correspond to early endosomes (Fig. 6A). To further evaluate the ability of FTY720 to induce LAT1-GFP endocytosis, we subjected cells to cell-surface biotinylation and incubated them with FTY720 for various times. After cleavage of the remaining cell-surface biotin, the intracellular biotinylated proteins were purified and immunoblotted in order to detect LAT1-GFP (Fig. 6B). We observed a clear increase in the amount of internalized LAT1-GFP when FTY720 was present, confirming that the drug stimulates LAT1 endocytosis.

We also used fluorescence microscopy to locate LAT1-GFP in cells incubated for longer times in the presence of FTY720 (Fig. 6C). After 6 h of treatment, LAT1-GFP was largely located at the surface of large intracellular compartments that were not labelled by the LAMP1 late endosomal/lysosomal marker. These compartments resembled the vacuoles recently observed in cells treated with FTY720 or with the analog SH-BC-893, shown to be enlarged multivesicular bodies (MVBs). Formation of these vacuoles is due to another effect of FTY720, namely mislocalization of the PI3P 5-kinase PIKfyve, which regulates membrane fusion and formation of internal vesicles of MVBs^16^. That LAT1-GFP reached these enlarged MVBs in FTY720-treated cells further illustrates that LAT1-GFP endocytosis is stimulated under these conditions. In yeast, FTY720 did not impair proper targeting of internalized permeases to the vacuole, suggesting that the late endocytic pathway is not impaired by FTY720.

### FTY720 elicits reduction of LAT1/SLC7A5 activity and mTORC1 inhibition

**Figure 7:**
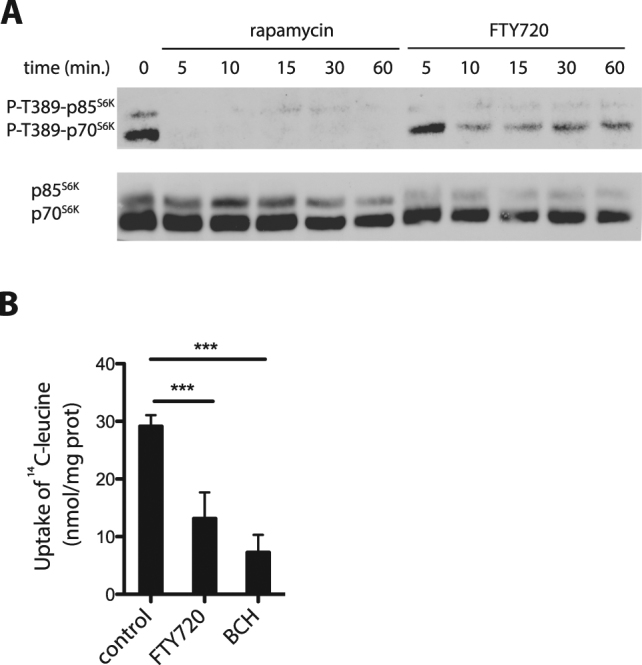
FTY720 affects mTORC1 signaling and LAT1 activity. (**A**) T-REx HeLa cells were incubated with rapamycin (200 ng/ml) or FTY720 (5 μM) for the indicated times. Cell extracts were immunoblotted with anti-p70^S6K^/p85^S6K^ and anti-phospho-p70^S6K^/p85^S6K^ (Thr389). (**B**) HeLa cells were incubated for 3 min in the presence of 0.1 mM [^14^C]-leucine alone or 0.1 mM [^14^C]-leucine with 5 μM FTY720 or 2 mM BCH. Data are means of four independent experiments and error bars correspond to standard deviations (unpaired *t*-test, ***P < 0.001).

As LAT1 is known to play an important role in mTORC1 activation^12^ because of its major contribution to leucine transport^15^, we sought to determine whether mTORC1 is inhibited in FTY720-treated HeLa cells and whether this might correlate with decreased LAT1 activity. We thus examined by western blot analysis the phosphorylation status of the p70S6K and p85S6K kinases. Rapamycin-treated HeLa cells were used as a control. FTY720 was found to cause a significant reduction of p70S6K and p85S6K phosphorylation (Fig. 7A). This effect was most pronounced after a 10-min incubation of the cells in the presence of FTY720. As expected, inhibition of mTORC1 by rapamycin was more pronounced and faster. We next examined whether FTY720 also inhibits the inherent activity of LAT1 (Fig. 7B). HeLa cells were incubated for 3 min in the presence of [^14^C]-leucine alone, [^14^C]-leucine and FTY720, or [^14^C]-leucine and 2-amino-2-norbornanecarboxylic acid (BCH), a competitive inhibitor of L-type amino acid transporters. As expected, HeLa cells were found to incorporate leucine at a high rate, and the presence of BCH reduced this uptake. The presence of FTY720 also significantly reduced the uptake of leucine. This indicates that the intrinsic activity of LAT1, and possibly that of other transporters contributing to leucine uptake, is inhibited in the presence of FTY720. These results suggest that in HeLa cells as in yeast, added FTY720 rapidly reduces the intrinsic activity of transporters such as LAT1/SLC7A5, and that this causes inhibition of mTORC1 signaling.

## Discussion

**Figure 8:**
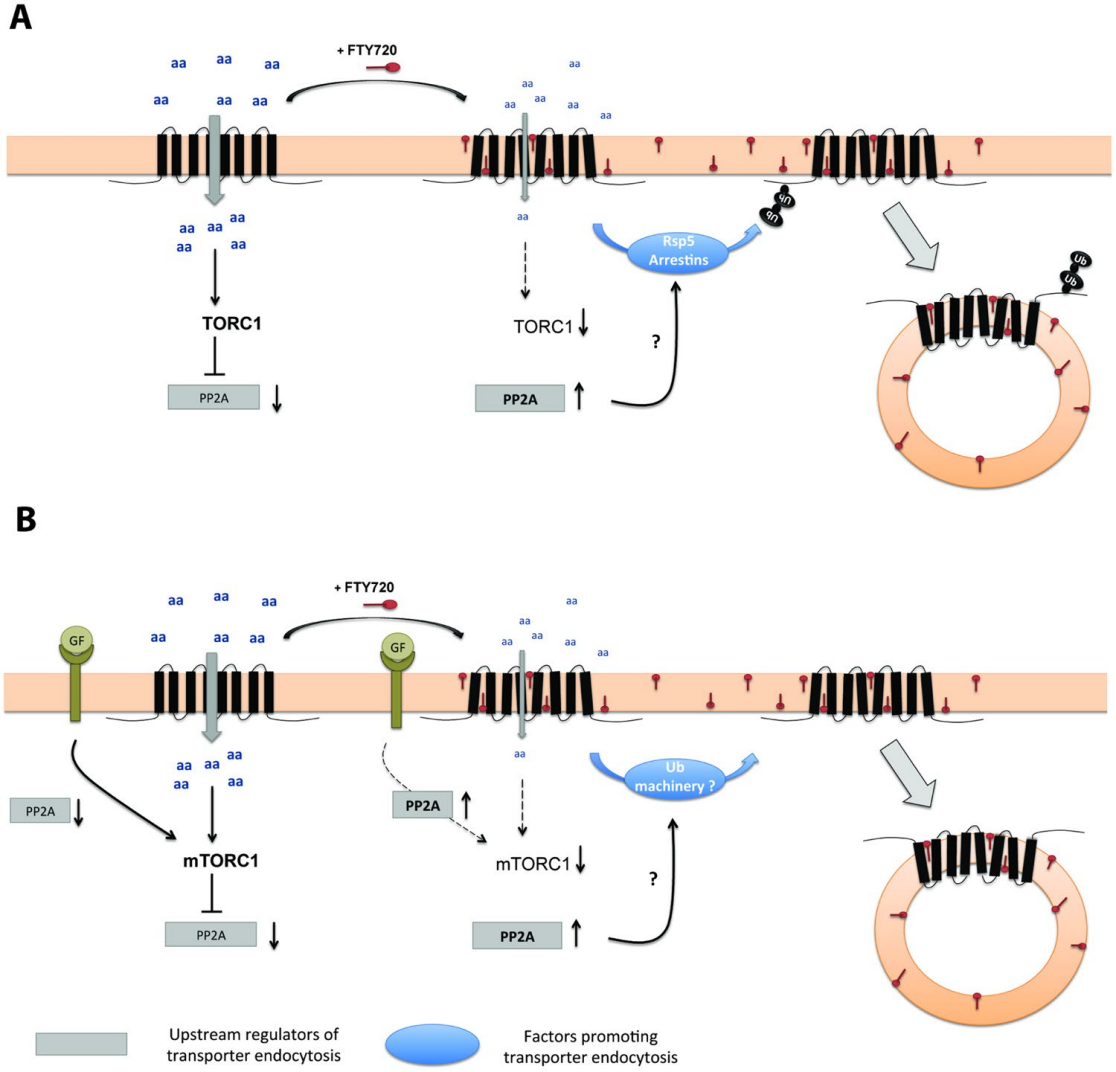
Model of FTY720-induced transporter endocytosis in yeast and human cells. (**A**) In yeast, exogenously supplied FTY720 molecules insert into the plasma membrane, thereby causing a decrease of the intrinsic activity of nutrient transporters, including amino acid permeases. The resulting decrease of nutrient uptake causes inhibition of the TORC1 kinase complex. This elicits activation of an unknown mechanism which then somehow stimulates the ability of the α-arrestin-family adaptor proteins and the Rsp5 ubiquitin ligase to promote permease ubiquitylation, triggering permease sorting into the endocytic pathway. (**B**) In mammalian cells, mTORC1 is regulated by nutrient availability and growth factors (GF). Addition of FTY720 similarly alters the inherent activity of nutrient transporters, including LAT1/SLC7A5, thereby causing mTORC1 inhibition. This reduced mTORC1 signaling contributes to stimulation of a mechanism involving a PP2A phosphatase which can activate a machinery, possibly dependent on ubiquitin, which promotes the endocytosis of nutrient transporters, including LAT1/SLC7A5. The signaling pathway responding to GFs also influences the cell-surface stability of nutrient transporters by regulating both mTORC1 and the PP2A-dependent mechanism under mTORC1 control. The presence of FTY720 inhibits both GF-dependent functions (via a mechanism involving a PP2A phosphatase), thereby contributing further to stimulation of transporter endocytosis.

Our study suggests that the mechanisms underlying transporter endocytosis in response to FTY720 treatment are in several respects similar in yeast and human cells. We discuss below each aspect of this complex cellular reaction, and propose a tentative unifying model which also emphasizes the particularities of each system (Fig. 8).

In yeast, we have found FTY720 to cause rapid reduction of the activity of several plasma membrane permeases. In cells where endocytosis is impaired, we have demonstrated unambiguously that what is altered is the inherent activity of these proteins. In the case of Gap1, our more detailed analysis shows that its V_max_ is reduced in the presence of FTY720, while its *K*_m_ remains normal. Our observations on HeLa cells are similar: we have found FTY720 to reduce the transport activity of LAT1, and the rapidity of this effect again suggests that the drug acts by reducing the intrinsic activity of the protein rather than by causing its internalization into endocytic vesicles. How exogenously supplied FTY720 inhibits the intrinsic activity of transporters remains unknown. As FTY720 chemically resembles sphingoid bases, it likely behaves like natural sphingoid bases, integrating first into the plasma membrane before being internalized into the cell. The natural sphingoid base of yeast phytosphingosine (PHS) acts as a precursor for synthesizing phytoceramide at the level of the endoplasmic reticulum (ER). Phytoceramide then migrates to the Golgi, where it is converted to complex sphingolipids (SLs)^36^. Inhibition of the first step of SL synthesis can be compensated by exogenous supply of PHS. This means that PHS, after inserting into the plasma membrane, is internalized and reaches the ER. Consistently with the view that FTY720 uses the same transport pathway, fluorescent derivatives of FTY720 label the plasma membrane and the ER^37,38^. Initial insertion of FTY720 into the plasma membrane could alter the lipidic microenvironment and possibly the structure and activity of plasma membrane proteins, including transporters. If so, such effects can be expected when natural sphingoid bases are exogenously supplied to cells. PHS is indeed reported to reduce the uptake of uracil and several amino acids into yeast cells, and in strains auxotrophic for these compounds, this results in strong reduction of growth^39–41^. Furthermore, we have observed that PHS, like FTY720, induces efficient endocytosis of Gap1 and a rapid drop in Gap1^K9R,K16R^ intrinsic activity (Fig. S2). A comparison of the effects of PHS and similar compounds has further revealed that the amino group at position C-2 and the hydroxyl group at position C-4 are important features of PHS for efficient inhibition of membrane transport. It has therefore been proposed that these groups protrude out of the membrane and directly contact transmembrane proteins^40^. Interestingly, FTY720 displays amino and hydroxyl groups, both at position C-2, that could likewise be important in interfering with transporter function.

Our results show that another immediate consequence of FTY720 addition in both yeast and HeLa cells is strong reduction of TORC1 signaling. In Hela cells, mTORC1 inhibition is probably due to rapid FTY720-elicited inhibition of LAT1 activity, as LAT1 is well known to be the main leucine transporter in most cancer cells^42^, and leucine is not only an essential amino acid and the most abundant amino acid in proteins, but also a key regulator of mTORC1 signaling^15,43^. The essential role of LAT1 in mTORC1 signaling is illustrated by the rapid deactivation of mTORC1 in cells incubated with BCH, a competitive inhibitor of LAT1^12^. Although the loss of LAT1 activity in FTY720-treated cells could in principle be sufficient to explain the associated inhibition of mTORC1 signaling, additional mechanisms are most likely involved. In FTY720-treated Jurkat cells, for instance, the Akt kinase involved in mTORC1 stimulation by growth factors is inactivated by dephosphorylation^44^. The putative FTY720-elicited inactivation of additional nutrient transporters could also contribute to mTORC1 inhibition. In yeast, inhibition of TORC1 signaling upon FTY720 addition could likewise be a consequence of the reduced activity of multiple nutrient transporters present at the plasma membrane. This view is supported by the similar and relatively rapid rates at which Gap1 activity decreases and TORC1 is inhibited upon FTY720 addition, as compared to the slower rates of Gap1 ubiquitylation and endocytosis. Furthermore, we have ruled out the possibility that Gap1 inactivation upon FTY720 addition might be due to reduced mTORC1 signaling, since rapamycin does not alter the activity of the Gap1^K9R,K16R^ mutant.

In yeast, TORC1 inhibition by rapamycin is known to promote downregulation of multiple plasma membrane transporters via the Rsp5 Ub ligase and adaptors of the α-arrestin family^28,29,45,46^. This bulk endocytosis of transporters followed by their degradation in the vacuole is thought to allow cells to retrieve amino acids, as does autophagy (which is also stimulated upon TORC1 inhibition). Furthermore, various stress conditions also promote endocytosis of multiple transporters, and the inhibition of TORC1 which typically occurs under these conditions is also suspected of contributing to this downregulation^29^. In the case of Gap1, the downregulation induced by rapamycin or stress conditions is elicited by ubiquitylation involving Rsp5 and the Bul1/2 and Aly1/2 α-arrestins^29^. This pathway of Gap1 ubiquitylation differs from those elicited by addition of ammonium or by the conformational changes coupled to transport catalysis^24,25^. Our study shows that FTY720-induced Gap1 ubiquitylation and endocytosis also involve the stress-responsive Bul1/2 and Aly1/2 α-arrestins. Although more complex models cannot be ruled out, the simplest interpretation of this observation is that Gap1 downregulation in FTY720-treated cells results solely from inhibition of TORC1 signaling. This model is supported by the fact that TORC1 inactivation occurs in the first few minutes after FTY720 addition and clearly precedes Gap1 ubiquitylation and endocytosis.

In human cells, the factors that promote sorting of LAT1 into the endocytic pathway remain poorly known. One possibility worth considering is an involvement of ARRDC proteins and Nedd4-type Ub ligases (orthologous, respectively, to yeast α-arrestins and Rsp5)^22^. Consistently with this view, a recent study has shown that Nedd4-2 silencing in human primary trophoblast (HPT) cells increases LAT1 activity and the LAT1 protein level^47^. As LAT1 is covalently linked to 4F2hc^48^, the cytosolic signals mediating LAT1-4F2hc endocytosis might be exposed on one or both of these two proteins. It is thus interesting to mention a study suggesting an important role of Ub in 4F2hc downregulation^49^. The upstream mechanisms controlling LAT1-4F2hc endocytosis in response to FTY720 seem more complex than in yeast. A contribution of mTORC1 inhibition is likely, as murine embryonic fibroblasts (MEFs) show much less pronounced FTY720-elicited endocytosis of 4F2hc when mTORC1 is hyperactive^50^. Earlier studies, however, have shown that mTORC1 inhibition by rapamycin is not sufficient to induce 4F2hc endocytosis^51^, and we have similarly failed to detect significant LAT1 internalization in rapamycin-treated HeLa cells (data not shown). It thus seems that rapamycin-insensitive mechanisms are able to promote cell surface accumulation of LAT1-4F2hc. In murine hematopoietic cells whose growth no longer depends on an exogenous supply of interleukin-3, rapamycin treatment has been shown, interestingly, to trigger efficient endocytosis of transporters, as in yeast^52^. Similarly, rapamycin causes a reduction of LAT1 activity in HPT cells, but it has no effect when mTORC1 is first stimulated by incubating these cells with insulin and IGF-I^53^. These observations again illustrate that mTORC1 may play a role in stabilizing LAT1-4F2hc. They also indicate that two pathways might in fact promote accumulation of LAT1-4F2hc at the plasma membrane, one dependent on mTORC1 and sustained by growth factors and nutrients, and another dependent on growth factors but independent of mTORC1, or at least insensitive to rapamycin. According to this view, FTY720 would interfere negatively with both pathways (Fig. 8).

Previous studies have shown that FTY720 stimulates the activity of PP2A phosphatases required to promote transporter endocytosis^8,44^. This property of FTY720 is shared with ceramides, which also stimulate the endocytosis of 4F2hc^54^. It is thus tempting to envisage that activated PP2A might play a role in the control of factors that mediate the endocytosis of LAT1-4F2hc and other transporters. Interestingly, this view is reminiscent of the proposed role of yeast Sit4/Cdc55/Ptd3, a PP2A complex modulated by TORC1, in permease ubiquitylation and endocytosis, via dephosphorylation of α-arrestins^24^. In yeast, furthermore, externally supplied ceramide is known to stimulate this PP2A complex and to inhibit growth^55^. These observations suggest an attractive possibility: that in yeast, stimulation of PP2A phosphatases in response to TORC1 inhibition contributes to FTY720-induced endocytosis of permeases (Fig. 8). To assess this model, we have tried to determine whether Sit4 might play a role in rapamycin-induced downregulation of Gap1. A lack of Sit4 did result in detectable protection against downregulation, reminiscent of the situation in human cells, but the result remains uncertain because the effect was visible in only a fraction of the cells, perhaps because *sit4* mutant cells grow very slowly (data not shown).

In summary, similarities clearly exist between the mechanisms responsible in human and yeast for FTY720-induced transporter endocytosis. Additional work is needed to better understand how the Bul/Aly/Rsp5 ubiquitylation machinery is stimulated upon TORC1 inactivation and to test whether PP2A phosphatases are involved. In human cells, the factors promoting LAT1-4F2hc endocytosis need to be better characterized. This would open the interesting prospect of investigating how the function of these factors is controlled by upstream mechanisms sensitive to mTORC1, growth factors, and FTY720.

## Methods

### Human cell culture and transfection

HeLa cells were grown in DMEM with 10% fetal bovine serum (FBS) and 1% penicillin/streptomycin. T-REx HeLa cells expressing LAT1-GFP were maintained under an atmosphere of 5% CO_2_ in MEM Glutamax supplemented with 10% FBS, 1% penicillin/streptomycin, 5 µg/ml blasticidin, and 200 μg/ml zeocin. Transcription of the LAT1-GFP gene in T-REx HeLa cells was induced by supplementing the medium with tetracycline (1 μg/mL) for 24 hours.

### Generation of a stable cell line

To generate stable cell lines expressing LAT1-GFP, T-REx HeLa cells were transfected with plasmid pCB001, a pcDNA4/TO/Myc-His B vector encoding hLAT1 fused to eGFP via a Gly-Ala linker. The vector also contains a 2 μ sequence and the *URA3* selection marker, allowing it to be used for constructions by recombination in yeast. Forty-eight hours after transfection, cells were transferred for 3 weeks to a selection medium including 5 µg/ml blasticidin and 200 μg/ml zeocin. Individual clones were isolated, expanded, and grown in MEM Glutamax containing 10% FBS, blasticidin, zeocin, and 1% penicillin/streptomycin. After expansion, single colonies were tested for expression of LAT1-GFP by addition of 1 μg/ml tetracycline for 24 h and subsequent analysis by western blotting and fluorescence microscopy.

### Yeast strains, growth conditions, and plasmids

All yeast strains used in this study (Table 1) derive from strain ∑1278b. Cells were grown at 29 °C under agitation in minimal buffered medium, pH 6.1^56^. The main carbon source was galactose (Gal) (3%) or glucose (Glu) (3%). In the experiments focusing on Gap1, Can1, or Lyp1, the nitrogen source present in the growth media was proline (10 mM) and in those focusing on Fur4, it was ammonium (Am) in the form of (NH_4_)_2_SO_4_ (20 mM). The plasmids used in this study are listed in Table 2. FTY720 (Cayman Chemical) dissolved in DMSO was added to yeast cultures to 10 μM final concentration.

**Table 1:** Yeast strains used in this study.

Strain	Genotype	Source or reference
23344c	*ura3*	Lab collection
27038a	*npi1-1 ura3*	20
EK008	*gap1Δ ura3*	Lab collection
35101a	*gap1Δ aly1Δ aly2Δ ura3*	29
JA493	*gap1Δ bul1Δ bul2Δ ura3*	25
MA062	*gap1Δ aly1Δ aly2Δ bul1Δ bul2Δ ura3*	29
MA025	*gap1Δ BUL1-FLAG bul2Δ ura3*	24
MA032	*gap1Δ BUL2-HA ura3*	24
CJ005	*gap1Δ npi1-1 ura3*	Lab collection
35288a	*gap1Δ can1Δ npi1-1 ura3*	This study

**Table 2:** Plasmids used in this study.

Plasmid	Description	Source or reference
pFL38	*CEN-ARS* (*URA3*)	60
pJOD10	*CEN-ARS-GAL1-GAP1-GFP* (*URA3*)	59
pCJ038	*CEN-ARS GAL1-GAP1* ^*K9R,K16R*^ *-GFP* (*URA3*)	61
pCJ004	*CEN-ARS GAL1-GAP1* (*URA3*)	Lab collection
pCJ563	*CEN-ARS GAL1-CAN1-GFP* (*URA3*)	25
pNAM001	*CEN-ARS LYP1-GFP* (*URA3*)	58
pFL38-gF-GFP	*CEN-ARS-GAL1-FUR4-GFP* (*URA3*)	Lab collection
pRS426-GST	*pRS426-pADH-GST* (*URA3*)	62
pRS426-GST-Bmh2	*pRS426-pADH-GST-BMH2* (*URA3*)	62
pl436	*YEp-SCH9-HA* (*URA3*)	33
pCJ555	*YEp-CMVp-GFP- stop* (*TetR*) (*URA3*)	This study
pET-41a(+)-hLAT1	*pET-41a*(+)-*GST-hLAT1*	63
pCB001	*YEp-CMVp-hLAT1-*(*GA*)*5*- *GFP* (*Tet R*) (*URA3*)	This study

### Permease activity assays

The transport activities of Gap1 Can1, Lyp1, and Fur4 were determined, respectively, by measuring incorporation of [^14^C]-citrulline, [^14^C]-arginine, [^14^C]-lysine, or [^14^C]-uracil as previously described^57^. The apparent *K*_m_ and V_max_ values of Gap1^K9R, K16R^ were determined by measuring incorporation of [^14^C]-citrulline added at 1, 5, 25, 50, 75, 100, 200, and 300 μM^58^. Uptake of [^14^C]-leucine into HeLa cells was measured on cells grown for 48 h to reach 70–85% confluence. The cells were then washed twice with Na^+^-free HBSS containing choline-Cl (137 mM) and Hepes (10 mM), and incubated for 3 min at 37 °C with 0.1 mM [^14^C]-leucine −/+ 5 μM FTY720 or 2 mM BCH. Thereafter, the cells were placed on ice, washed three times with cold HBSS, and lysed by incubation for 20 min with 200 μl of 100 mM NaOH. The cell lysate was added to a scintillation tube and radioactivity was counted in a Beckman scintillation counter.

### Fluorescence microscopy analysis of yeast

For the subcellular localization of Gap1-GFP, Can1-GFP, and Lyp1-GFP, cells were first grown exponentially in galactose-proline medium. Glucose was then added for 90 min to repress expression of the permease gene. For the localization of Fur4-GFP, cells were grown on raffinose-ammonium, galactose was added for 1 h, and then glucose was added for an additional hour. In some experiments, cells were also incubated with 7-amino-4-chloromethylcoumarin (CMAC) to stain the vacuole. Cells were immobilized on a thin layer of 1% agarose and viewed at room temperature with a fluorescence microscope (Nikon Eclipse 80i) equipped with a 100x differential interference contrast NA 1.40 Plan- Apochromat objective (Nikon) and appropriate fluorescence light filter sets. Images were captured with a digital camera (Nikon, DS-Qi1Mc) and processed with ImageJ and Adobe Illustrator.

### Immunofluorescence analysis of T-REx HeLa cells

10^5^ cells were seeded on coverslips in 24-well plates. Expression of LAT1-GFP was induced by treatment for 24 h with 1 µg/ml tetracycline. The cells were then washed and grown for 24 h in a tetracycline-free medium. They were fixed for 15 min with 3% paraformaldehyde, permeabilized for 3 min with 0.1% Triton X-100, and incubated for 15 min with 0.5 M NH_4_Cl to reduce free aldehyde groups. Nonspecific staining was blocked for 30 min with fetal calf serum (10%) in phosphate-buffered saline (PBS). When indicated, cells were stained with a mouse primary antibody against Lamp1 (Abcam, ab25630, 1/250x) followed by a goat anti-mouse secondary antibody. DAPI was used for nuclear staining. The coverslips were mounted on microscope slides and the cells were viewed with an inverted confocal microscope (Zeiss LSM 710 with a 63X/1.4 objective) and processed with ImageJ and Adobe Illustrator.

### Live cell imaging of T-REx HeLa cells

Cells were seeded on CELLview cell culture dishes. The expression of LAT1-GFP was induced by treatment for 24 h with tetracycline (1 µg/ml). After 24 h, the medium was replaced with DMEM without tetracycline and the cells were transfected with a plasmid encoding Rab5-mCherry. For this, Lipofectamine 2000 was used according to the manufacturer’s instructions. After 24 h, the transfection medium was replaced with DMEM/F-12 containing HEPES and lacking phenol red. Forty hours after transfection, the cells were placed in an incubation chamber pre-heated at 37 °C and visualized with an inverted confocal microscope (Zeiss LSM 710 with a 63X/1.4 objective). Images were processed with ImageJ and Adobe Illustrator.

### Image quantification

Colocalization of LAT1-GFP and Rab5-mCherry is reported using Pearson’s correlation coefficient. A region of interest was manually drawn around each cell and Pearson’s correlation coefficient was calculated with the plug-in Coloc_2 (Fiji) for at least 32 cells. Pearson’s correlation coefficient is presented in box-and-whisker plots. Prism software was used to assess the statistical significance of the data with the unpaired *t-*test after using the D’Agostino-Pearson test to test the normality of the distribution.

### Protein extracts, western blotting and GST pulldown

For western blot analysis of T-REx HeLa cell extracts, cells were lysed with cold NP40 buffer (150 mM NaCl, 1% NP40, 50 mM Tris-Hcl pH 8) supplemented with a protease inhibitor cocktail (Roche, n°04693159001). An equal volume of 2x loading buffer (500 mM Tris Base, 50 mM Tris HCl pH 6.8, 2 mM EDTA, 2% SDS, 10% glycerol, 0.01% bromophenol blue, and 2% β-mercaptoethanol) was added to the cell lysate and the solution was heated for 5 min at 99 °C. For western blot analysis of yeast proteins, crude cell extracts were prepared as previously described^20^. After transfer to a nitrocellulose membrane (Schleicher and Schuell), proteins were probed with mouse anti-GFP (Roche, catalog nb 11814460001, dilution 1/10000x), anti-hemagglutinin (anti-HA) (12CA5; Roche; dilution 1/5000x), anti-P-T737-Sch9 (1/2500) (Saliba *et al*. in preparation), anti-FLAG (F1804; Sigma, dilution 1/2000), or anti-LAT1 (abcam, ab85226, dilution 1/1000x). Primary antibodies were detected with horseradish-peroxidase-conjugated anti-mouse or anti-rabbit immunoglobulin G secondary antibody (GE Healthcare, 1/10000x). Bound antibodies were revealed by chemiluminescence (Roche, 12015196001). In the glutathione S-transferase (GST) pulldown experiments, exponentially growing cells were harvested before and 30 min after addition of ammonium (50 mM) or 60 min after addition of FTY720 (10 μM). GST pulldown was carried out as described previously^24^. Proteins were analyzed by immunoblotting with anti-GST (Invitrogen, dilution 1/10000) or anti-FLAG antibodies.

### Limited proteolysis

This experiment was carried out as previously described^34^. Briefly, cells were treated or not with 10 μM FTY720 for 30 min and then lysed with glass beads in 200 μl buffer (50 mM HEPES pH 7.5, 300 mM NaCl, with or without FTY720). A total membrane fraction was generated by centrifuging at 100,000 × g for 60 min in a SW55 Ti rotor (Beckman Coulter, Fullerton, CA). Membranes were resuspended in buffer and incubated at a trypsin:protein ratio of 1:5. Samples were incubated with trypsin (0.1 mg/ml) for 0, 0.5, 2, 5, 10, and 30 min and the reaction was stopped by adding trypsin inhibitor type I-S from soybean. A control reaction was run for 30 min in the absence of trypsin. Proteins were precipitated by adding 10% trichloroacetic acid (TCA), and Gap1-GFP was analyzed by western blotting with anti-Gap1^59^ and anti-GFP antibodies.

### Cell-surface biotinylation

A 6-well plate was first seeded with 5 × 10^5^ cells in each well. Expression of LAT1-GFP was induced by a 24 h treatment with tetracycline (1 µg/ml). Cells were washed and incubated for an additional 40 h in tetracycline-free medium. The cells were washed twice with PBS+ (1 mM MgCl_2_, 0.1 mM CaCl_2_) and incubated with sulfo-NHS-biotin for 30 min at 4 °C. They were then incubated at either 4 °C (to block membrane trafficking) or 37 °C for various times in the presence or absence of FTY720 (5 µM). Next the cells were washed in PBS+ and the remaining surface biotin was cleaved by two 20-min incubations with Mesna buffer (50 mM Tris pH 8.8, 100 mM NaCl, 50 mM Mesna, 0.2% FBS) to reduce its disulfide bond. Then the cells were incubated for 20 min in quenching buffer (50 mM Tris pH 8.8, 100 mM NaCl, 50 mM iodoacetamide, 0.2% FBS) followed by two washes with PBS+ . The cells were lysed with NP40 buffer, and biotinylated proteins were purified by affinity chromatography using streptavidin-coated beads. LAT1-GFP was then detected by immunoblotting with anti-GFP antibody.

### Statistical analyses

Statistical analyses were performed using Prism 5 software. Unpaired *t*-test was used to calculate *P*-values. Error bars on graphs show the standard deviation.

### Data availability

All data generated and analyzed during this study are included in this published article (and its Supplementary Information files). Additional data corresponding to negative results are available from the corresponding author on request.

## Electronic supplementary material

Supplementary Information

## References

[1] Adachi K (1995). Design, synthesis, and structure-activity relationships of 2-substituted-2-amino-1,3-propanediols: Discovery of a novel immunosuppressant, FTY720. Bioorg. Med. Chem. Lett..

[2] Brinkmann V (2010). Fingolimod (FTY720): discovery and development of an oral drug to treat multiple sclerosis. Nat Rev Drug Discov.

[3] Brinkmann V (2002). The immune modulator FTY720 targets sphingosine 1-phosphate receptors. J. Biol. Chem..

[4] Mandala S (2002). Alteration of lymphocyte trafficking by sphingosine-1-phosphate receptor agonists. Science.

[5] Gräler MH, Goetzl EJ (2004). The immunosuppressant FTY720 down-regulates sphingosine 1-phosphate G-protein-coupled receptors. FASEB J..

[6] Matloubian M (2004). Lymphocyte egress from thymus and peripheral lymphoid organs is dependent on S1P receptor 1. Nature.

[7] Brinkmann V, Cyster JG, Hla T (2004). FTY720: sphingosine 1-phosphate receptor-1 in the control of lymphocyte egress and endothelial barrier function. Am. J. Transplant..

[8] Romero Rosales K (2011). Sphingolipid-based drugs selectively kill cancer cells by down-regulating nutrient transporter proteins. Biochem. J..

[9] Selwan, E., Finicle, B. T., Kim, S. M. & Edinger, A. L. Attacking the supply wagons to starve cancer cells to death. *FEBS Lett*. n/a–n/a, 10.1002/1873-3468.12121 (2016). 10.1002/1873-3468.12121 PMC4833639 26938658

[10] Kanai Y (1998). Expression cloning and characterization of a transporter for large neutral amino acids activated by the heavy chain of 4F2 antigen (CD98). J. Biol. Chem..

[11] Yanagida O (2001). Human L-type amino acid transporter 1 (LAT1): characterization of function and expression in tumor cell lines. Biochim. Biophys. Acta.

[12] Nicklin P (2009). Bidirectional transport of amino acids regulates mTOR and autophagy. Cell.

[13] Sinclair LV (2013). Control of amino-acid transport by antigen receptors coordinates the metabolic reprogramming essential for T cell differentiation. Nat. Immunol..

[14] Hansen, C. G., Ng, Y. L. D., Lam, W.-L. M., Plouffe, S. W. & Guan, K.-L. The Hippo pathway effectors YAP and TAZ promote cell growth by modulating amino acid signaling to mTORC1. *Cell Res*., 10.1038/cr.2015.140 (2015). 10.1038/cr.2015.140 PMC4670996 26611634

[15] Nagamori S (2016). Structure-activity relations of leucine derivatives reveal critical moieties for cellular uptake and activation of mTORC1-mediated signaling. Amino Acids.

[16] Kim SM (2016). Targeting cancer metabolism by simultaneously disrupting parallel nutrient access pathways. J. Clin. Invest..

[17] Chalfant CE (1999). Long chain ceramides activate protein phosphatase-1 and protein phosphatase-2A. Activation is stereospecific and regulated by phosphatidic acid. J. Biol. Chem..

[18] Welsch CA, Hagiwara S, Goetschy JF, Movva NR (2003). Ubiquitin pathway proteins influence the mechanism of action of the novel immunosuppressive drug FTY720 in Saccharomyces cerevisiae. J. Biol. Chem..

[19] Lauwers E, Erpapazoglou Z, Haguenauer-Tsapis R, André B (2010). The ubiquitin code of yeast permease trafficking. Trends Cell Biol..

[20] Hein C, Springael JY, Volland C, Haguenauer-Tsapis R, André B (1995). *NPl1*, an essential yeast gene involved in induced degradation of Gap1 and Fur4 permeases, encodes the Rsp5 ubiquitin-protein ligase. Mol. Microbiol..

[21] Rotin D, Kumar S (2009). Physiological functions of the HECT family of ubiquitin ligases. Nat. Rev. Mol. Cell Biol..

[22] Becuwe M, Herrador A, Haguenauer-Tsapis R, Vincent O, Léon S (2012). Ubiquitin-mediated regulation of endocytosis by proteins of the arrestin family. Biochem. Res. Int..

[23] MacGurn JA, Hsu P-C, Emr SD (2012). Ubiquitin and membrane protein turnover: from cradle to grave. Annu. Rev. Biochem..

[24] Merhi A, André B (2012). Internal amino acids promote Gap1 permease ubiquitylation via TORC1/Npr1/14-3-3-dependent control of the Bul arrestin-like adaptors. Mol. Cell. Biol..

[25] Ghaddar K (2014). Substrate-induced ubiquitylation and endocytosis of yeast amino Acid permeases. Mol. Cell. Biol..

[26] Guiney, E. L., Klecker, T. & Emr, S. D. Identification of the endocytic sorting signal recognized by the Art1-Rsp5 ubiquitin ligase complex. *Mol. Biol. Cell* **27**, mbc.E16–08–0570–4054 (2016). 10.1091/mbc.E16-08-0570 PMC5156545 27798240

[27] Becuwe M (2012). A molecular switch on an arrestin-like protein relays glucose signaling to transporter endocytosis. J. Cell Biol..

[28] Zhao Y, MacGurn JA, Liu M, Emr S (2013). The ART-Rsp5 ubiquitin ligase network comprises a plasma membrane quality control system that protects yeast cells from proteotoxic stress. Elife.

[29] Crapeau M, Merhi A, André B (2014). Stress conditions promote yeast Gap1 permease ubiquitylation and down-regulation via the arrestin-like Bul and Aly proteins. J. Biol. Chem..

[30] Gournas C, Amillis S, Vlanti A, Diallinas G (2010). Transport-dependent endocytosis and turnover of a uric acid-xanthine permease. Mol. Microbiol..

[31] Keener JM, Babst M (2013). Quality control and substrate-dependent downregulation of the nutrient transporter Fur4. Traffic.

[32] Crapeau, M. *et al*. A yeast Gap1 permease that fails to be ubiquitylated and downregulated in the presence of its substrates provokes PKA-mediated degradation of the Uga35(Dal81) transcription factor.

[33] Urban J (2007). Sch9 is a major target of TORC1 in Saccharomyces cerevisiae. Mol. Cell.

[34] Lauwers E, Grossmann G, André B (2007). Evidence for coupled biogenesis of yeast Gap1 permease and sphingolipids: essential role in transport activity and normal control by ubiquitination. Mol. Biol. Cell.

[35] Napolitano L (2017). Novel insights into the transport mechanism of the human amino acid transporter LAT1 (SLC7A5). Probing critical residues for substrate translocation. Biochim. Biophys. Acta.

[36] Megyeri M, Riezman H, Schuldiner M, Futerman AH (2016). Making Sense of the Yeast Sphingolipid Pathway. J. Mol. Biol..

[37] Ettmayer P (2006). NBD-labeled derivatives of the immunomodulatory drug FTY720 as tools for metabolism and mode of action studies. Bioorg. Med. Chem. Lett..

[38] Wickramasinghe, D., Timerman, R., Bartusek, J. & Heikal, A. A. In *Advanced Time-Correlated Single Photon* Counting *Applications* (ed. Becker, W.) **111**, 339–355 (Springer International Publishing, 2015).

[39] Skrzypek MS, Nagiec MM, Lester RL, Dickson RC (1998). Inhibition of amino acid transport by sphingoid long chain bases in Saccharomyces cerevisiae. J. Biol. Chem..

[40] Chung N, Mao C, Heitman J, Hannun YA, Obeid LM (2001). Phytosphingosine as a specific inhibitor of growth and nutrient import in Saccharomyces cerevisiae. J. Biol. Chem..

[41] Welsch CA, Roth LWA, Goetschy JF, Movva NR (2004). Genetic, biochemical, and transcriptional responses of Saccharomyces cerevisiae to the novel immunomodulator FTY720 largely mimic those of the natural sphingolipid phytosphingosine. J. Biol. Chem..

[42] Wang Q, Holst J (2015). L-type amino acid transport and cancer: targeting the mTORC1 pathway to inhibit neoplasia. Am J Cancer Res.

[43] Wolfson, R. L. *et al*. Sestrin2 is a leucine sensor for the mTORC1 pathway. *Science* aab2674, 10.1126/science.aab2674 (2015). 10.1126/science.aab2674 PMC4698017 26449471

[44] Matsuoka Y, Nagahara Y, Ikekita M, Shinomiya T (2003). A novel immunosuppressive agent FTY720 induced Akt dephosphorylation in leukemia cells. Br. J. Pharmacol..

[45] Beck T, Schmidt A, Hall MN (1999). Starvation induces vacuolar targeting and degradation of the tryptophan permease in yeast. J. Cell Biol..

[46] Jones CB (2012). Regulation of membrane protein degradation by starvation-response pathways. Traffic.

[47] Rosario, F. J., Dimasuay, K. G., Kanai, Y., Powell, T. L. & Jansson, T. Regulation of Amino Acid Transporter Trafficking by mTORC1 in Primary Human Trophoblast cells is Mediated by the Ubiquitin Ligase Nedd4-2. *Clin. Sci*. CS20150554 10.1042/CS20150554 (2015). 10.1042/CS20150554 PMC5681479 26608079

[48] Nakamura E (1999). 4F2 (CD98) heavy chain is associated covalently with an amino acid transporter and controls intracellular trafficking and membrane topology of 4F2 heterodimer. J. Biol. Chem..

[49] Eyster CA (2011). MARCH ubiquitin ligases alter the itinerary of clathrin-independent cargo from recycling to degradation. Mol. Biol. Cell.

[50] Guenther GG (2013). Loss of TSC2 confers resistance to ceramide and nutrient deprivation. Oncogene.

[51] Edinger AL (2007). Controlling cell growth and survival through regulated nutrient transporter expression. Biochem. J..

[52] Edinger AL, Thompson CB (2002). Akt maintains cell size and survival by increasing mTOR-dependent nutrient uptake. Mol. Biol. Cell.

[53] Rosario FJ, Kanai Y, Powell TL, Jansson T (2013). Mammalian target of rapamycin signalling modulates amino acid uptake by regulating transporter cell surface abundance in primary human trophoblast cells. J. Physiol. (Lond.).

[54] Guenther GG (2008). Ceramide starves cells to death by downregulating nutrient transporter proteins. Proc. Natl. Acad. Sci. USA.

[55] Nickels JT, Broach JR (1996). A ceramide-activated protein phosphatase mediates ceramide-induced G1 arrest of Saccharomyces cerevisiae. Genes Dev..

[56] Jacobs P, Jauniaux JC, Grenson M (1980). A cis-dominant regulatory mutation linked to the argB-argC gene cluster in Saccharomyces cerevisiae. J. Mol. Biol..

[57] Grenson M, Mousset M, Wiame JM, Béchet J (1966). Multiplicity of the amino acid permeases in *Saccharomyces cerevisiae*. I. Evidence for a specific arginine-transporting system. Biochim. Biophys. Acta.

[58] Ghaddar K (2014). Converting the yeast arginine Can1 permease to a lysine permease. J. Biol. Chem..

[59] De Craene JO, Soetens O, André B (2001). The Npr1 kinase controls biosynthetic and endocytic sorting of the yeast Gap1 permease. J. Biol. Chem..

[60] Bonneaud N (1991). A family of low and high copy replicative, integrative and single-stranded *S. cerevisiae*/*E. coli* shuttle vectors. Yeast.

[61] Lauwers E, André B (2006). Association of yeast transporters with detergent-resistant membranes correlates with their cell-surface location. Traffic.

[62] Mayordomo I, Regelmann J, Horak J, Sanz P (2003). Saccharomyces cerevisiae 14-3-3 proteins Bmh1 and Bmh2 participate in the process of catabolite inactivation of maltose permease. FEBS Lett..

[63] Napolitano L (2015). LAT1 is the transport competent unit of the LAT1/CD98 heterodimeric amino acid transporter. Int. J. Biochem. Cell Biol..

