# Supplementary material for: FTY720-induced endocytosis of yeast and human amino acid transporters is preceded by reduction of their inherent activity and TORC1 inhibition
Source: Sci Rep. 2017 Oct 23;7:13816. doi: 10.1038/s41598-017-14124-2 (PMC5653847; doi:10.1038/s41598-017-14124-2)
Supplement: Supplementary file 1 — Supplementary Information [file 41598_2017_14124_MOESM1_ESM.pdf]

Supplementary information

FTY720-induced endocytosis of yeast and human amino acid transporters is preceded by reduction of their inherent activity and TORC1 inhibition

Céline Barthelemy, Abdoulaye Oury Barry, Laure Twyffels, and Bruno André

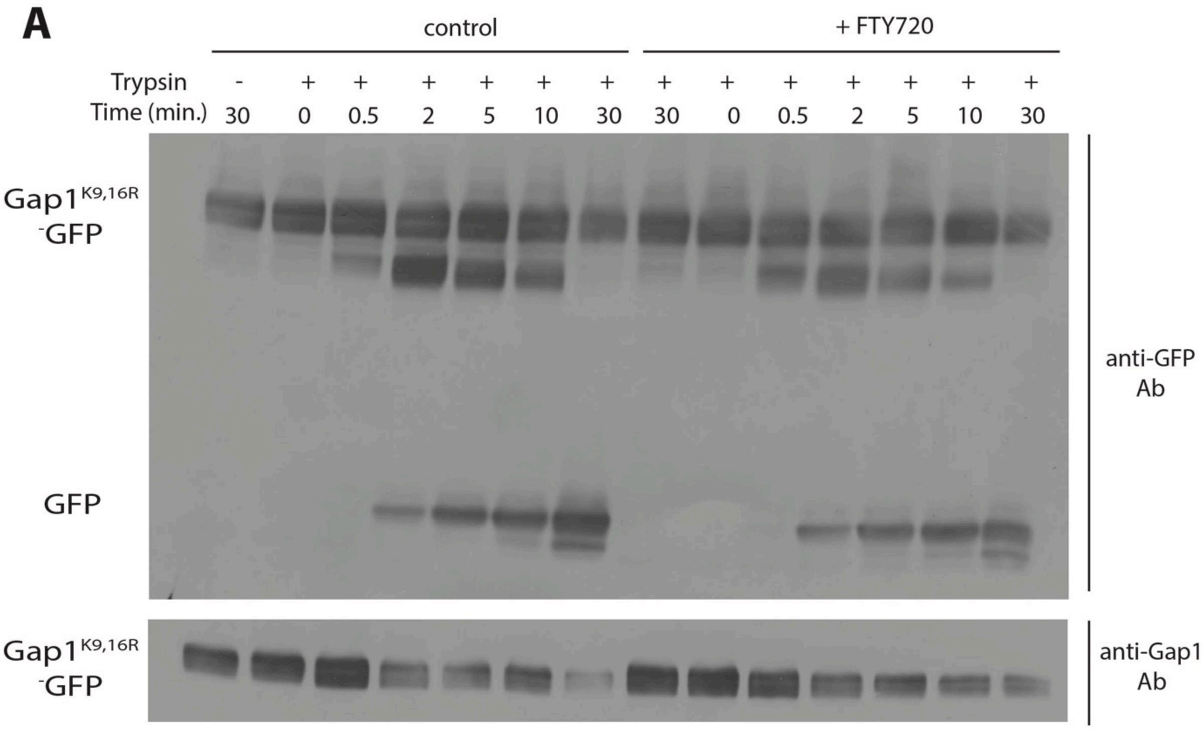

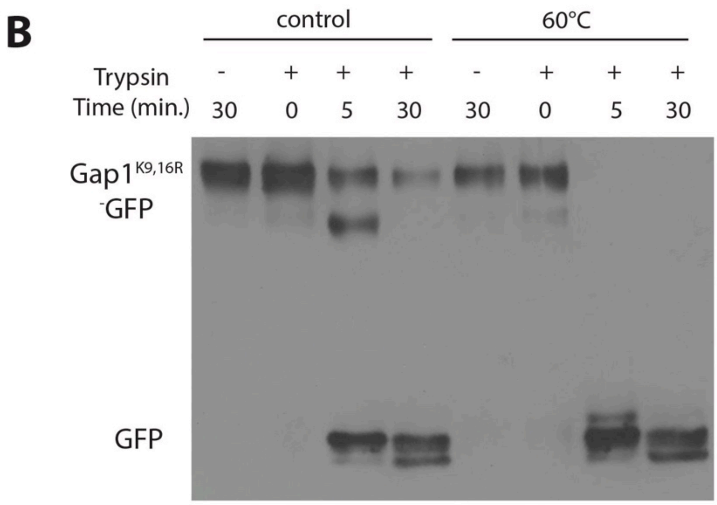

Figure S1. Figure S1. Influence of FTY720 on the sensitivity of Gap1(K9R,K16R) to limited proteolysis. A. Strain EK008 (*gap1Δ ura3*) transformed with plasmid pCJ038 (YCpGAL-GAP1^{K9R,K16R}-GFP) was grown on galactose-proline medium. Glucose was added for 1 h to inhibit Gap1 synthesis, and the cells were treated with 10 µM FTY720 or the solvent DMSO (control) for 30 min. Cell extracts were prepared. Trypsin was added or not and incubation was carried out for the indicated times before immunoblotting with anti-GFP and anti-Gap1 antibodies. B. Control experiment. Strains and experimental conditions as in A, except that the sensitivity of Gap1(K9R,K16R) to limited proteolysis was examined after heating the samples (at 60°C) or not for 4 min before trypsinolysis.

A

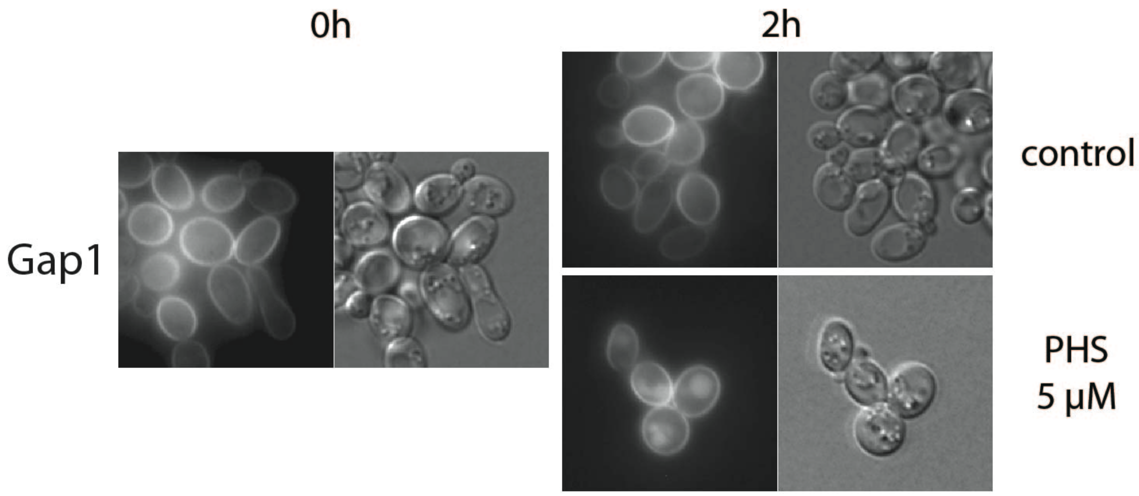

B

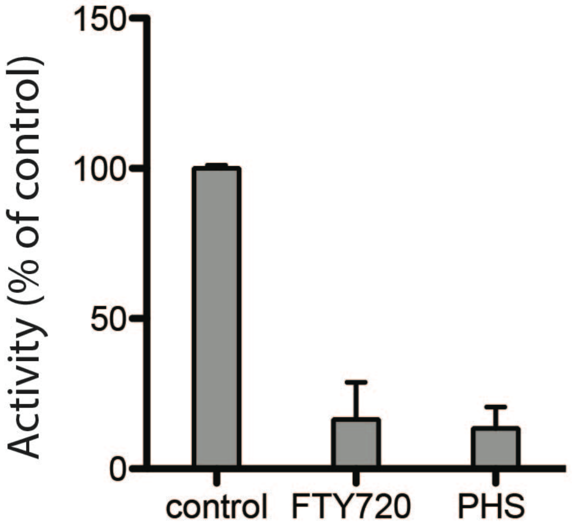

Figure S2. Phytosphingosine (PHS) affects the localization and intrinsic activity of Gap1. A. Strain 23344C (*ura3*) transformed with plasmid pJOD10 (YCpGAL-GAP1-GFP) was grown on galactose proline medium. Glucose was added for 90 min before addition of phytosphingosine (5 µM) or solvent alone (control). Cells were examined by epifluorescence microscopy before and 2 h after PHS addition. B. Strain EK008 (*gap1Δ ura3*) transformed with pCJ038 (YCpGAL-GAP1^{K9R,K16R}-GFP) was grown on galactose-proline medium. Glucose was added for 90 min to repress Gap1 synthesis. Cells were treated for 60 min with 10 µM FTY720 or 5 µM PHS. The initial uptake rate of [^{14}C]-labeled citrulline (75 µM), reflecting Gap1 activity, was then measured.

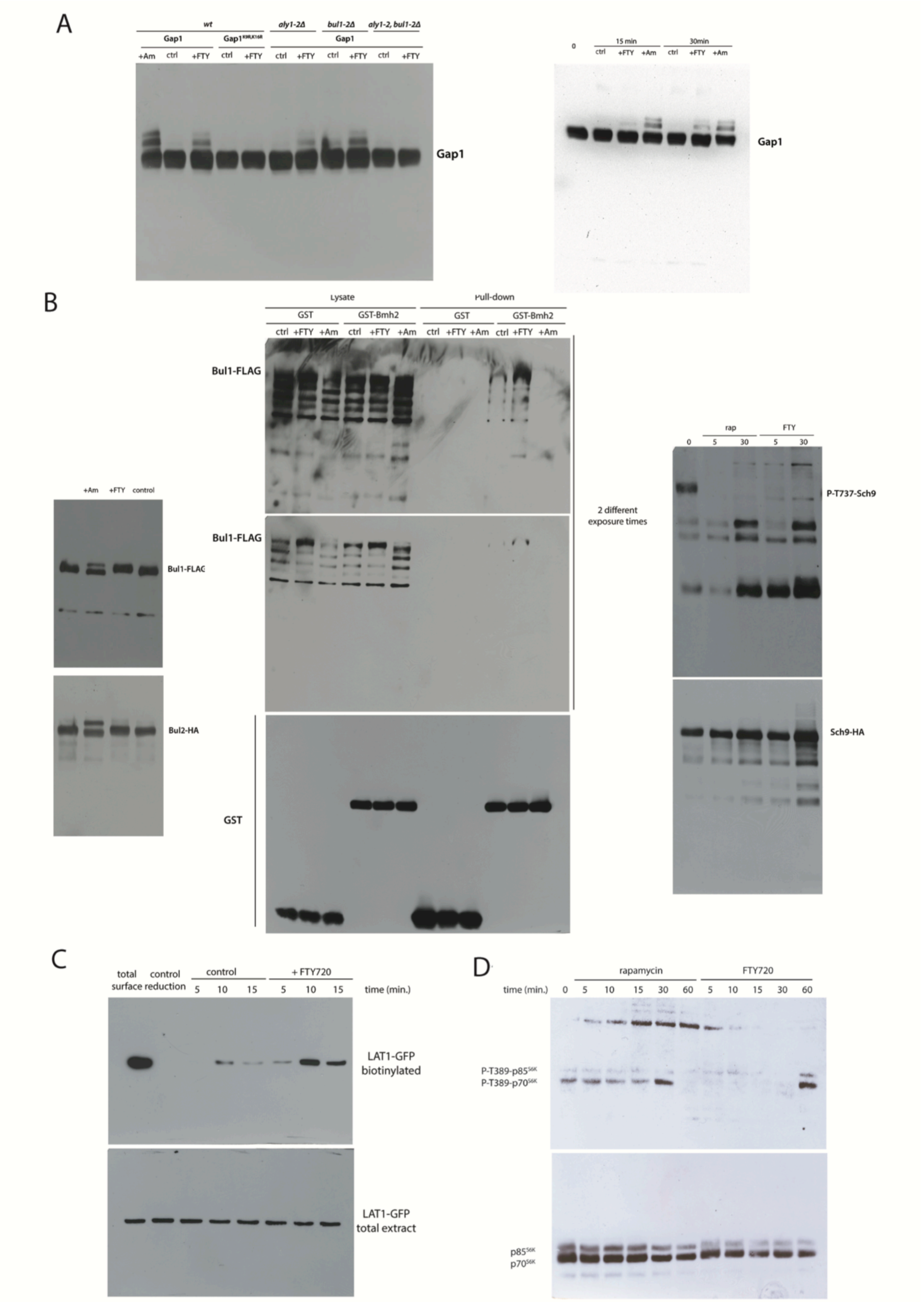

Figure S3. Full-length blots. Non-cropped versions of the blots presented in Figure 2 (A), Figure 3 (B), Figure 6 (C), and Figure 7 (D). The blot shown in Figure 5 has no tbeen cropped.
